# Disseminated primary diffuse leptomeningeal gliomatosis: a case report with liquid based and conventional smear cytology

**DOI:** 10.1186/1742-6413-2-16

**Published:** 2005-09-20

**Authors:** Masha Bilic, Cynthia T Welsh, Zoran Rumboldt, Rana S Hoda

**Affiliations:** 1 Department of Pathology & Laboratory Medicine, Medical University of South Carolina, Charleston, SC, USA; 2 Department of Radiology, Medical University of South Carolina, Charleston, SC, USA

**Keywords:** cytology, primary diffuse leptomeningeal gliomatosis, ventriculoperitoneal shunt, peritoneal metastasis

## Abstract

**Background:**

Primary diffuse leptomeningeal gliomatosis is a rare neoplasm confined to the meninges without evidence of primary tumor in the brain or spinal cord parenchyma. Cerebrospinal fluid diversion via ventriculoperitoneal shunt may be used as a therapeutic modality. Herein, we describe the first report of cytologic findings of a case of this neoplasm with shunt-related peritoneal metastasis.

**Case presentation:**

A 19-year-old male presented with a 6-month history of severe headaches. He had bilateral papilledema on physical exam. Cerebrospinal fluid examination was negative. Four months later a ventriculoperitoneal shunt was placed. Shortly thereafter, he was diagnosed with primary diffuse leptomeningeal gliomatosis based on the biopsy of an intradural extramedullary lesion adjacent to the lumbar spinal cord at a referral cancer center. The histology featured an infiltrating growth pattern of pleomorphic astrocytes with diffuse positivity for glial fibrillary acidic protein. A couple of months later he presented at our institution with ascites and an anterior peritoneal mass. Repeat cerebrospinal fluid cytology and fine needle aspiration of the mass confirmed disseminated gliomatosis. Cytologic characteristics included clusters of anaplastic cells of variable size, high nuclear to cytoplasm ratio and scant to moderate cytoplasm. Occasional single bizarre multinucleated cells were seen with eccentric "partial wreath-like" nuclei, clumped chromatin and prominent nucleoli. Patient expired 13 months after initial presentation.

**Conclusion:**

Disseminated primary diffuse leptomeningeal gliomatosis should be considered in the differential diagnosis of chronic aseptic meningitis and in the presence of a peritoneal tumor in patients with ventriculoperitoneal shunts. Immunocytochemistry may be of diagnostic value.

## Introduction

Primary diffuse leptomeningeal gliomatosis (PDLG) is a rare uniformly fatal neoplastic condition. It is characterized by widespread infiltration of the meninges by tumor thought to arise from heterotopic glial cell nests, without evidence of primary tumor within the brain or spinal cord parenchyma [1]. Glial heterotopia is defined as nests or linear arrays of glioneuronal tissue in the meninges. While their pathogenesis remains debatable, such heterotopic nests have been noted in the subarachnoid space in about 1% of unselected autopsies [2]. The incidence is substantially higher in patients with congenital anomalies, particularly those of the central nervous system [2,3]. Neoplastic transformation of extramedullary heterotopic glial tissue is a rare event, usually diagnosed at autopsy. One of the earliest reports of this entity was in 1936 by Bailey [4]. Since then, fewer than two dozen cases overall, and fewer than ten cases predominantly affecting the meninges around the spinal cord have been reported in the English language literature [1].

Patients with PDLG most often present with symptoms and signs of intracranial hypertension, such as headache and papilledema. Examination of the cerebrospinal fluid (CSF) characteristically shows raised opening pressure, elevated protein concentration and lymphocyte pleocytosis. Diagnosis is often delayed mainly because of the nonspecific nature of the symptoms and CSF findings, such that empirical antituberculous therapy is frequently tried in patients with PDLG [1,5]. There appears to be no cure, however, variable clinical and radiologic remissions lasting up to 4 years from presentation have been achieved utilizing aggressive chemotherapeutic and radiation regimens [1,6,7]. Cytologic examination of CSF in PDLG is most often negative. One series reported only 1 of 8 cases of PDLG having "atypical cells" on CSF cytology, without further elaboration of cytologic findings [8]. This is in contrast to a positive antemortem CSF cytologic diagnosis in 8 out of 12 patients with secondary meningeal spread from intracranial malignant glioma [9].

To reduce the intracranial pressure, some patients undergo a CSF diversion procedure in the form of ventriculo-peritoneal (VP) or alternate route shunting [5,6]. Peritoneal tumor dissemination by way of VP shunt is a known complication in patients with different primary central nervous system (CNS) tumors, most often germinomas [10,11]. According to our review of the literature, this complication has not been previously reported in PDLG.

Herein we describe the cytologic features of CSF and fine needle aspiration (FNA) of PDLG with VP shunt-related peritoneal spread.

## Case Report

A 19 year old college freshman presented, in January of 2004, with a 6 month history of severe recurring global headaches. He had no past medical or travel history. Physical examination was notable for bilateral papilledema. His workup included magnetic resonance imaging (MRI) of the brain and intracranial vessels, which initially did not reveal any abnormalities. Initial examination of the CSF revealed an opening pressure of 50 cm H_2_O (normal: 10–20 cm), protein of 778 mg/dL (normal: 15–45 mg/dL), glucose of 44 mg/dL (normal: >45 mg/dL), an absolute WBC count of 24/mm^3 ^with 55% lymphocytes and 45% macrophages. A full microbiology workup was negative for organisms. The CSF was negative by polymerase chain reaction (PCR) for enteroviruses and West Nile virus. Flow cytometry showed no evidence of lymphoma or leukemia. Cytologic examination of the CSF was negative for malignant cells, showing predominantly reactive monocytes. The patient was initially managed symptomatically with analgesics and diuretics. By May of 2004, he developed cranial nerve palsies necessitating placement of a VP shunt. The patient sought a medical second opinion at another institution, where a diagnostic biopsy was obtained of an intradural extramedullary lesion at T12/cauda equina via vertebral laminectomy.

**Figure 1:**
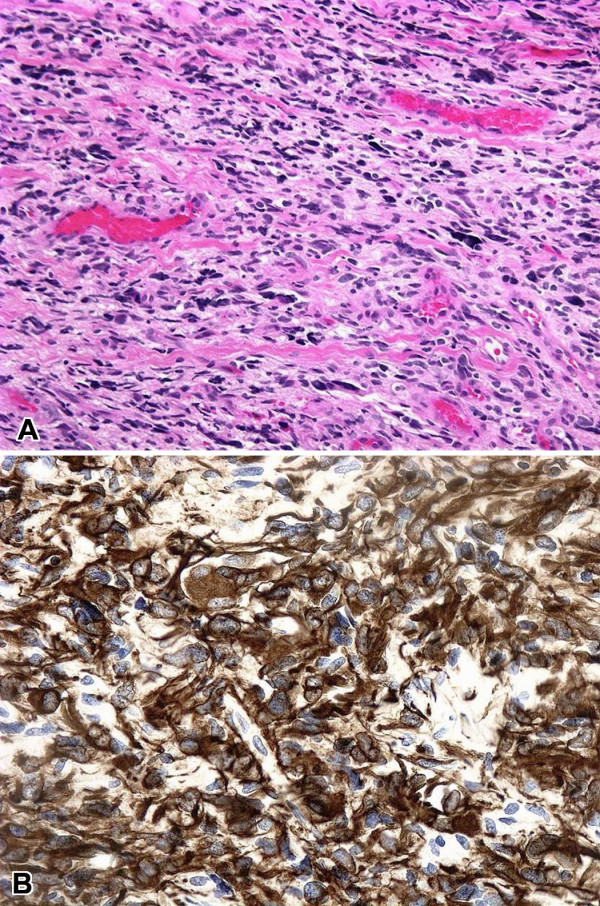
**A. **Biopsy of an intradural extramedullary lesion at T12/cauda equina, showing highly pleomorphic cells with a hint of fibrillary cytoplasm growing in an infiltrating fashion (H&E, ×100). **B. **Diffuse cytoplasmic staining of tumor cells with antibody for GFAP (GFAP, ×200).

**Figure 2:**
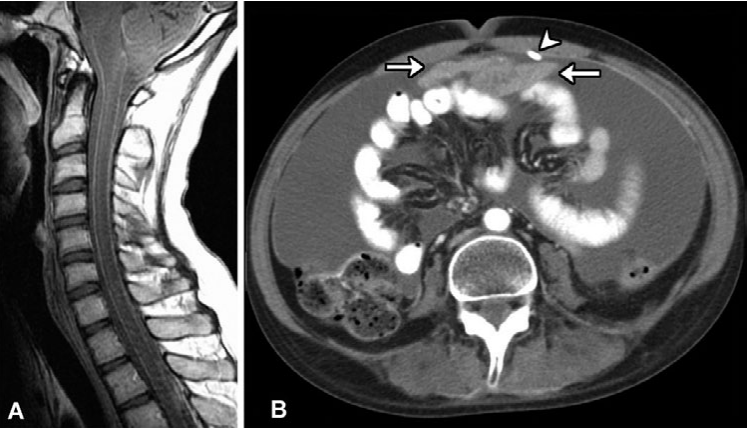
**A. **Postcontrast sagittal T1-weighted MR image of the cervical spine shows diffuse leptomeningeal enhancement along the surface of the spinal cord. **B. **Axial contrast-enhanced CT image through the abdomen demonstrates extensive ascites and an enhancing midline mass (arrows) adjacent to the shunt catheter (arrowhead).

The biopsy showed an infiltrating growth pattern of pleomorphic astrocytes. The nuclei were hyperchromatic with irregular borders. Blood vessels were numerous. Dense, wavy collagen bands were noted throughout the tumor, and seemed particularly apparent in the vicinity of blood vessels (Figure 1A). Necrosis and psammoma bodies were absent. The neoplastic cells had variable amounts of fibrillary cytoplasm which was diffusely positive for glial fibrillary acidic protein (GFAP) (*DakoCytomation*, Carpinteria, CA, 1:3000 dil; Figure 1B). A diagnosis of glial neoplasm consistent with leptomeningeal gliomatosis was made. Subsequently, the patient returned for care at our institution. At this time, MRI showed peripheral contrast enhancement of the meninges surrounding the entire spinal cord (Figure 2A) as well as prominent thickening and enhancement of the cauda equina, consistent with diffuse leptomeningeal disease. Brain MRI study showed only subtle leptomeningeal and subarachnoid spread, without evidence of a primary tumor. He received aggressive craniospinal axis radiation and temozolomide chemotherapy which resulted in clinical improvement for a period of about two months. By August of 2004 the patient developed bilateral lower extremity paraplegia and recurrent seizures. Five months later, he developed massive ascites and an anterior peritoneal mass (Figure 2B). A computed tomography (CT)-guided FNA of the peritoneal mass was performed. The FNA specimen was processed as air-dried Diff-Quik (DQ) stained and alcohol-fixed Papanicolaou (Pap) stained direct smears, one ThinPrep (TP) slide and a cell block. A diagnosis of VP shunt-related peritoneal spread of PDLG was made.

**Figure 3:**
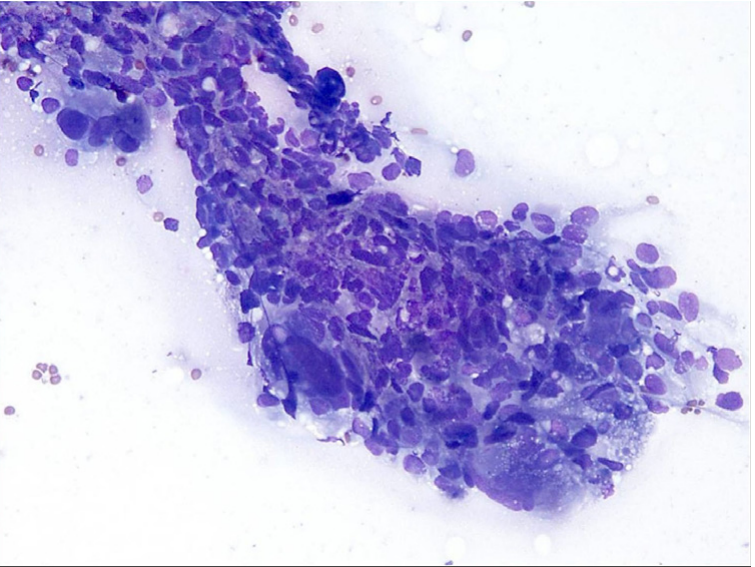
CT-guided FNA of anterior peritoneal mass, showing a somewhat cohesive large cluster of highly pleomorphic cells (Diff-Quik, ×200).

**Figure 4:**
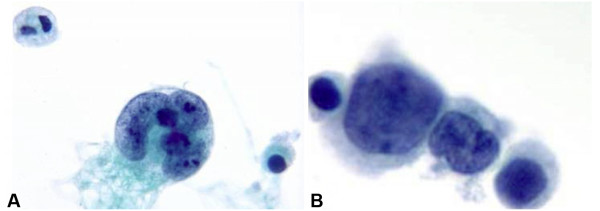
Cytomorpholgy of primary diffuse leptomeningeal gliomatosis involving the peritoneum. **A. **Single bizzare multinucleated neoplastic cells display "partial wreath-like" nuclear configuration, coarse chromatin clumping and multiple nucleoli (TP, Pap stain, ×1000). **B. **Clusters of smaller tumor cells showing variation in size, high nuclear to cytoplasm ratio, scant homogeneous cytoplasm, with even chromatin distribution and occasional clefting of the nuclear membrane (TP, Pap stain, ×1000).

DQ-stained smears showed pleomorphic malignant cells in poorly formed, somewhat cohesive clusters with haphazard cellular arrangement. The nuclei were hyperchromatic with great variation in size. Cytoplasmic borders were indistinct (Figure 3). The TP showed scattered single bizarre, very large multinucleated neoplastic cells with "partial wreath-like" or "horse shoe" nuclear configuration, coarse chromatin clumping, prominent nucleoli, and fibrillary cytoplasmic tails (Figure 4A). Clusters or nests of smaller, mononuclear neoplastic cells were also seen featuring high nuclear to cytoplasm ratio, scant homogeneous cytoplasm, occasional nuclear clefting and more even chromatin distribution (Figure 4B). Scattered cytoplasmic GFAP positivity was present in the cell block material from ascites collected 3 days prior to the FNA. Core biopsy of the peritoneal mass was confirmatory.

**Figure 5:**
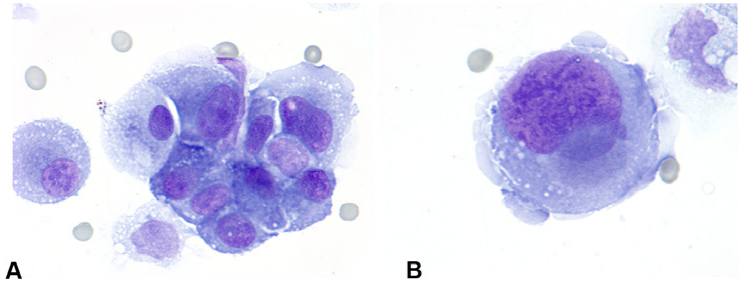
CSF cytology. **A. **Nests of anaplastic tumor cells are seen with round to oval eccentric nuclei, coarse chromatin, prominent nucleoli and moderate homogeneous cytoplasm (Diff-Quik, ×1000). **B. **Single giant malignant cells with similar morphology are also seen (Diff-Quik, ×1000).

A repeat CSF specimen was processed as two Cytospin slides, one stained with DQ and one with Pap. It showed a cytomorphology similar to that of the peritoneal mass FNA (Figures 5A and 5B).

The patient continued to deteriorate clinically. In addition to the non-improving neurologic status, he developed bowel obstruction which was unresponsive to palliative pelvic radiation and peritoneal infusion of phosphorus (P32) radioisotope. He expired in early February 2005, 13 months after initial presentation. An autopsy was declined.

## Discussion

Primary diffuse leptomeningeal gliomatosis (PDLG) is a rare neoplastic disorder limited to the meninges, in the absence of primary tumor within the brain or spinal cord parenchyma [1]. The diagnosis is most often made at autopsy, mainly because of lack of specific clinical, radiologic and laboratory diagnostic criteria, and the progressive natural history of the disease [1,5,8,10]. As such, appropriate management of affected patients is often delayed [1,5]. Despite rare encouraging reports of clinical improvement after aggressive radiation and chemotherapy [6,7], the disease is fatal in the majority of cases [1,5,6,8]. The survival depends, in part upon the World Health Organization (WHO) differentiation grade of tumor, complicating lesions (e.g. infarcts), and site of tumor [1].

The case presented in this report is unique in several respects. It describes an exceedingly uncommon disorder, PDLG, with peritoneal seeding as a result of symptomatic treatment (VP shunt). While this complication is known to occur in several different types of primary CNS tumors [10,11], such a clinical course has not been previously described in PDLG. This patient's tumor appeared poorly differentiated, befitting WHO grade III or IV. Such high tumor grade was likely a key factor determining aggressive tumor behavior, and also the relative ease with which it was recognized in various cytologic specimen preparations described above. A confirmatory piece of evidence in support of the final diagnosis was demonstration of GFAP-staining cells in ascitic fluid and core biopsy material of the peritoneal mass.

Immunocytochemistry has been advocated as a useful adjunct test in addition to routine CSF cytology in differentiating chronic aseptic meningitis from leptomeningeal carcinomatosis or gliomatosis especially in clinically suggestive cytologically negative cases [12]. It seems far fetched to suggest an evidence-based approach of indiscriminate GFAP immunocytochemistry in addition to the routine cytologic CSF examination. Nevertheless, the case presented here serves to remind us of the need to remain vigilant in complete review of patient information, forthcoming in communication with clinicians and other specialists, and diligent in formulation of differential diagnosis in each case. Only such an approach will offer patients standard-of-care treatments, and allow for improvements in those standards.
